# Ventricular Septal Rupture—The Resurgence of a Post-Myocardial Infarction Dreadful Complication during COVID-19 Pandemic

**DOI:** 10.1155/2023/3521526

**Published:** 2023-01-05

**Authors:** João Ferreira Reis, Luís Almeida Morais, Lídia Sousa, António Fiarresga

**Affiliations:** Department of Cardiology, Hospital de Santa Marta, Centro Hospitalar de Lisboa Central, Lisbon, Portugal

## Abstract

In the midst of the coronavirus disease-2019 (COVID-19) pandemic, an 84-year-old female patient was admitted due to non-exertional syncope preceded by retrosternal pain. She had experienced a prolonged episode of oppressive chest pain 6 days before her presentation, but due to the concern of contracting COVID-19, she did not present for medical care. Upon admission to the emergency department, the patient was in circulatory shock, with her physical examination being remarkable for the presence of a holosystolic murmur. Admission electrocardiogram revealed an inferior ST-segment elevation with Q waves with extension to the posterior wall, consistent with subacute infarct in the right coronary artery (RCA) territory, and the patient was transferred for primary percutaneous coronary intervention. Upon arrival to the catheterization laboratory, a summary transthoracic echocardiogram was performed, which revealed inferior wall and infero-septal akinesia with an 18 mm ventricular septal rupture. Coronary angiography documented occlusion of the proximal segment of a dominant RCA. Due to a high perioperative risk, the patient underwent successful retrograde percutaneous closure with a 24 mm MemoPart™ device, with mild to moderate residual shunt. Despite an immediate clinical improvement, the patient died 12 hours after the procedure due to refractory cardiogenic shock.

## 1. Introduction

The coronavirus disease-2019 (COVID-19) pandemic required a significant redeployment of healthcare resources and introduced major challenges to the management of life-threatening cardiovascular emergencies, such as ST-segment elevation myocardial infarction (STEMI). Several reports have suggested a medical care avoidance behaviour among patients with acute coronary syndrome (ACS) during the COVID-19 pandemic, leading to delayed ACS presentations and an increased rate of mechanical complications and, thus, a higher mortality [1–5].

Ventricular septal rupture (VSR) is a devastating complication following acute myocardial infarction (MI), and it is associated with a delayed presentation and/or late reperfusion [6]. The incidence of VSR has decreased from 1–3% following STEMI in the pre-reperfusion era to 0.17–0.31% following primary percutaneous coronary intervention (PPCI) [7]. One-month survival without intervention is of only 6%. Surgical repair may be required urgently, but there is no consensus on the optimal timing for surgery, with a 30-day mortality of 40% [8]. Early surgery is associated with a high perioperative mortality and concerns regarding tissue fragility and a high risk of recurrent ventricular rupture, while delayed surgery allows an easier definitive septal repair but carries the risk of rupture extension and death while waiting for surgery [8]. Thus, appropriate patient selection is of paramount importance. Percutaneous VSR closure (PVC) may be used as a temporizing measure to reduce shunt or as a bailout strategy for patients whose comorbidities preclude surgical repair [9].

## 2. Case Report

We present the case of an 84-year-old female patient with a past medical history of well-controlled arterial hypertension and polymyositis under corticosteroid therapy, who presents to the emergency department (ED) due to non-exertional syncope with associated retrosternal discomfort. She had experienced a prolonged episode of oppressive chest pain 6 days before her ED presentation, for what she called the prehospital medical assistance line, but due to the concern of contracting COVID-19, she did not accept being taken to a hospital facility. During the course of the next days, the patient had progressively less frequent intermittent episodes of chest pain.

**Figure 1:**
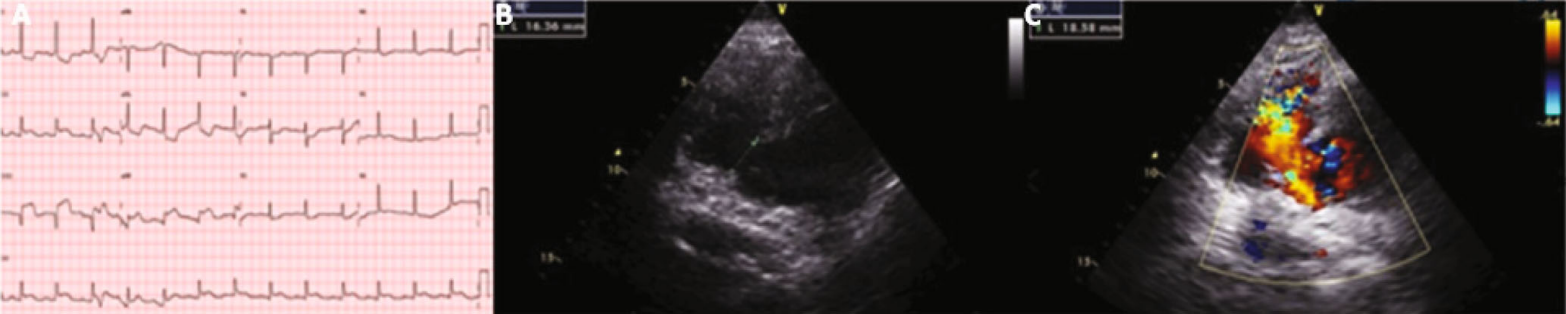
(a) ECG showing ST-segment elevation in the inferior leads with Q waves. (b) and (c) TTE showing a 18 mm VSR with left-to-right shunting by color-Doppler.

Upon arrival to the ED, the patient was found to be lethargic, hypotensive (blood pressure −70/40 mmHg), and tachypneic with a peripheral oxygen saturation of 92% while breathing room air and with signs of inadequate perfusion to the extremities. An electrocardiogram was promptly performed, which revealed inferior ST-segment elevation with Q waves, horizontal ST depression in V1-3, and reciprocal ST depression in lateral leads, compatible with inferoposterior STEMI (Figure 1(a)). Aspirin 300 mg, clopidogrel 600 mg, and unfractionated heparin 5000 IU were administered, and she was immediately referred to our Centre for PCI.

Once in the Catheterization Laboratory, a holosystolic murmur was heard across the precordium, and a transthoracic echocardiogram (TTE) was readily performed. It revealed mild left ventricular (LV) systolic dysfunction with inferior and infero-septal akinesia and right ventricular (RV) extension; an 18 mm VSR, with left-to-right shunting by Doppler color-flow imaging; and mild pericardial effusion (Figures 1(b) and 1(c)).

**Figure 2:**
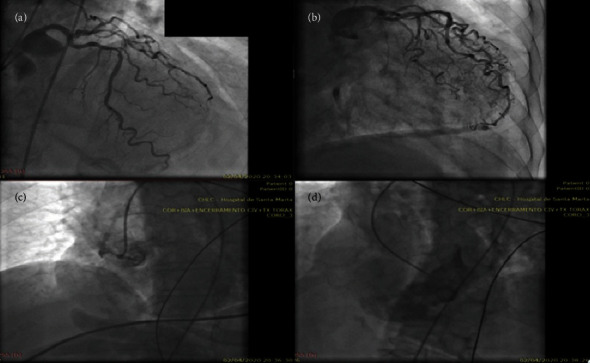
(a) and (b) ICA showing a 50% stenosis of the mid-segment of the LAD and a mid-segment occlusion of the RCA (c). Coronary collaterals are a prognostically important adaptive response in coronary artery disease. Note its absence in (c), which correlate with a larger final infarct volume and an increased risk of mechanical complications. (d) Ventriculography revealing the presence of a LV-to-RV shunt.

Invasive coronary angiography (ICA) documented proximal occlusion of dominant right coronary artery (RCA) and a 50% lesion of the mid-segment of the left anterior descending artery (LAD) (Figures 2(a), 2(b), and 2(c)). LV ventriculography showed the presence of a shunt with contrast passing from LV to RV (Figure 2(d)). The case was promptly discussed by a Multidisciplinary Heart Team, and the surgical risk was deemed prohibitive.

The patient evolved with circulatory collapse and neurological deterioration, requiring endotracheal intubation for airway protection. She received mechanical circulatory support (MCS) with intra-aortic balloon pump (IABP) in order to reduce afterload and, consequently, reduce the left-to-right shunt, and inotropic support was started.

**Figure 3:**
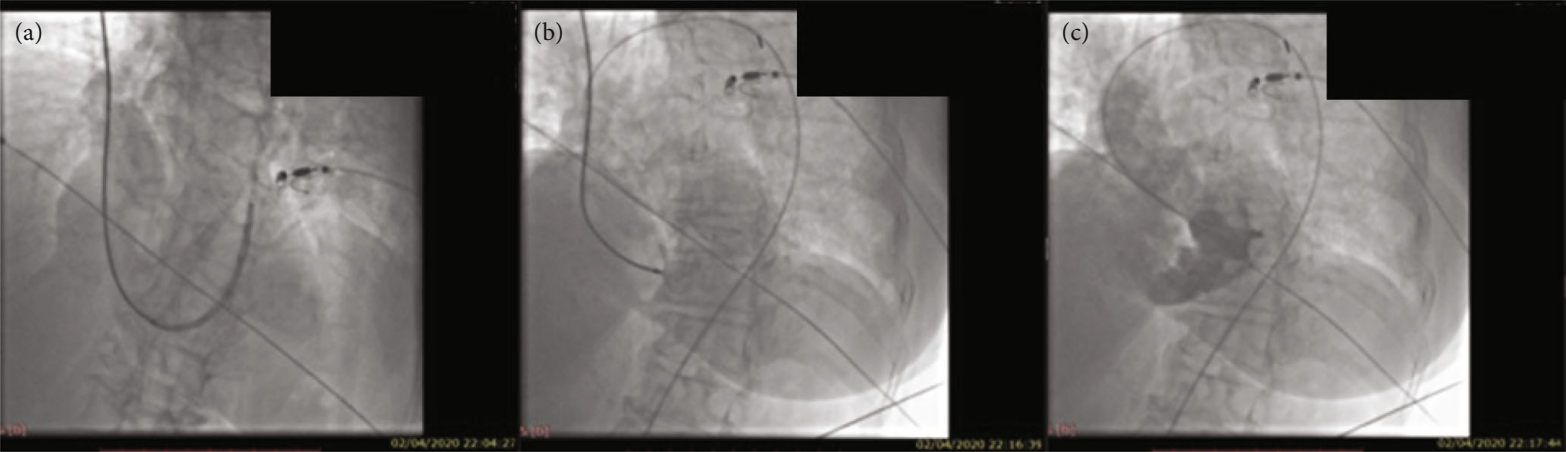
(a) and (b) Successful implantation of a 24 mm MemoPart™ device across the VSR. (c) Ventriculography showing mild to moderate residual shunt.

The right femoral artery and right internal jugular vein were cannulated, and the patient underwent PVC. A guidewire was introduced from the femoral artery, advanced through the VSR into the pulmonary artery, and a second snaring wire was introduced through the jugular vein to form an arteriovenous loop. The delivery sheath was advanced from the venous side loop over the guidewire through the VSR into the LV, and an atrial septal defect (ASD) occluder device 24 mm MemoPart™ was implanted (Figures 3(a) and 3(b)). LV angiogram and TTE confirmed a stable device position, with no interference with atrioventricular valve function and mild to moderate residual shunt (Figure 3(c)). The procedure was performed at nighttime, and dedicated ventricular septal defect closure devices were not available on the shelf.

Device implantation was associated with immediate improvement of the circulatory status, allowing temporary weaning from mechanical and inotropic support.

However, after an initial improvement, there was a progressive clinical deterioration, and the patient evolved with refractory cardiogenic shock and multi-organ failure despite high-dose vasopressors and maximal IABP support (1 : 1). She eventually died 12 h after the procedure.

## 3. Discussion

The COVID-19 pandemic has put healthcare systems worldwide under considerable pressure, making STEMI management increasingly challenging, due to a decrease in the number of patients presenting to hospitals, delays in emergency medical system transfer times, prolonged ED evaluations, delays in cardiac catheterization laboratory activation, and door-to-balloon time. Several reports reveal a 40% reduction in catheterization laboratory STEMI activations, during the early phase of the pandemic. A survey by the European Society of Cardiology indicates that the impact of COVID-19 on STEMI presentations is substantial, with both lower presentations (65.2% clinicians indicated a reduction in STEMI presentations >40%) and a higher rate of delayed presentations (60% clinicians reported that STEMI patients presented later than usual beyond the optimal window for PCI or thrombolysis) [10].

VSR after ACS is increasingly rare in the PCI era, but mortality remains high, and it has been shown that its incidence is lower in patients who undergo PPCI compared with those who undergo delayed or elective PCI after recent ACS [8]. There are recent case reports of VSR in patients who presented with delayed STEMI due to the constraints caused by the pandemic and were associated with a poor prognosis [11, 12]. It is recommended that all patients with ACS have a brief echocardiographic evaluation for mechanical complications prior to PPCI, and the presence of systolic murmur over the precordium should raise the suspicion of a VSR, especially in the presence of hemodynamic compromise.

Surgery remains the treatment of choice but is associated with an overall operative mortality of 42.9%, which varies significantly depending on its timing [8]. PVC is emerging as an alternative in inoperable patients or as salvage therapy for residual defects following surgical repair [8, 13]. Schlotter et al. found an overall procedure success rate of 89% and a 30-day or in-hospital mortality of 32% [9]; however, a 30-day mortality of 88% was described in those in circulatory shock [14].

The anatomical features of our patient's VSR, namely its size and location, posed technical challenges as defects >15 mm, and inferior/posterior defects are associated with increased procedural complexity [8], due to the lack of an adequate tissue ‘rim' to secure the device. However, the subacute nature of the ACS probably provided enough tissue stability to the device. Additionally, our patient met several factors proven to be associated with a worse outcome—older age, female gender, large defect size, lack of revascularization, cardiogenic shock—and was under steroid therapy [13]. The MCS provided by the IABP was crucial to stabilize the patient during the procedure by reducing left-to-right shunt and increasing coronary perfusion.

It would have been reasonable to provide full, more effective long-term MCS, such as ExtraCorporeal Membrane Oxygenation (ECMO), in order to stabilize hemodynamics, prevent end-organ injury, and safely allow to defer intervention or act as a bridge-to-decision. In a multidisciplinary evaluation, it was decided to not upscale MCS due to the patient's age and comorbidities.

Despite no clear evidence to guide the management of VSR patients, it is crucial that cardiologists are prepared to recognize the consequences of delayed STEMI presentation and are familiar with the possible management strategies.

## 4. Conclusions

This clinical case seeks to raise awareness to the potential complications of delayed medical care in STEMI during the pandemic. The fear of contracting COVID-19 infection will likely result in an increase in non-COVID morbidity and mortality, and the rate of STEMI-related mechanical complications is expected to increase. Percutaneous closure of a VSR is a feasible bailout procedure in selected high-risk patients and is associated with a high procedural success in experienced hands; however, appropriate patient selection and timing of the procedure are critical to maximize results.

## Additional Points

*Learning Objectives*. (1) To recognize that a timely and accurate diagnosis is a crucial factor in STEMI care, as delayed presentations of ACS are associated with increased rate of mechanical complications and a higher mortality. (2) To identify patients at high risk for STEMI-related mechanical complications, namely VSR, such as the presence of a late-presenting STEMI and signs of circulatory shock and/or a holosystolic murmur. (3) To understand the prognosis and the importance of a multidisciplinary approach in the management of post-MI VSR, recognizing percutaneous closure as a viable alternative to surgery in selected patients.
